# Radiochemical and analytical aspects of inter-institutional quality control measurements on radiopharmaceuticals

**DOI:** 10.1186/s41181-018-0052-1

**Published:** 2019-01-28

**Authors:** Erik de Blois, Rory M. S. de Zanger, Ho Sze Chan, Mark Konijnenberg, Wouter A. P. Breeman

**Affiliations:** 1 Department of Radiology and Nuclear Medicine, Erasmus MC, Wytemaweg 80, 3015CN, Post office box number: 2040, 3000 CA Rotterdam, The Netherlands; 2 Nano Instrumentation, TNO, Delft, The Netherlands; 3 AlfaRim Medical Holding BV, Delft, The Netherlands; 4 Department of Internal Medicine, Erasmus MC, Rotterdam, The Netherlands

**Keywords:** Standardization, Radiopharmaceuticals, HPLC, RCP, Arbitrary unit, Multicenter trial, Validated conditions

## Abstract

**Background:**

Clinically applied radiopharmaceuticals have to meet quality release criteria like a high radiochemical yield and radiochemical purity. Many radiopharmaceuticals do not have marketing authorization and have no dedicated monograph within the European pharmacopeia, therefore general monographs on quality control have to be applied for clinical applications. These criteria require standardization and validation in labeling and preparation, including QC measurements according to well-defined standard operation procedures. QC measurements however, are often based on detection techniques specific for a certain LC-system. Multi-institutional research and development of new radiopharmaceuticals lead to an increase in multicenter trials. Although all institutes’ radiopharmacies are using the same standardized labeling and operation procedures, they often use different LC and radiodetection systems. Here we present a comparison of QC assessments for 3 radiopharmaceuticals with focus on the interpretation of chromatograms, data-output and potential differences in local practical performances of QC on (U)HPLC.

**Methods:**

QC assessments for [^111^In]In-CCK, [^68^Ga]Ga-Bombesin and [^177^Lu]Lu-PSMA analogs were compared. Two of the radiopharmaceutical QC assessments were also applied in other institutes using their own HPLC-systems and concordant software. Data from the HPLC-injections and measurements is processed and summarized in chromatograms, based on a variety of smoothing algorithms for which different software programs are applied. Described radiopeptides were labeled and analyzed according their standardized labeling and operation procedures.

**Results:**

Integration of main peaks on chromatograms resulted in a range of RCP, depending on the smoothing algorithm used. [^111^In]In-CCK(A), ^68^Ga-Bombesin(B) and [^177^Lu]Lu-PSMA(C) analogs had a RCP range of 88%–96%(A), 89–95%(B) and 92–99%(C) respectively. Important factors affecting final RCP value were site specific background radiation-levels, intrinsic system properties such as noise and sensitivity, personal interpretation e.g. peak-tailing and smoothing algorithms.

**Conclusion:**

Measurement of RCP shows a strong method- and system-dependency, even when parameters are validated, standardized and SOP are followed. Release criteria are frequently based on RCP data from one central location. The lack of inter inter institutional validation and standardization in RCP determination makes the results therefore rather arbitrary. For multicenter trials, we recommend to compare locally determined RCP under validated and standardized conditions of in-line activity detection between institutes for each radiopharmaceutical.

## Introduction

### Background

In Nuclear Medicine, radiopharmaceutical Quality Control (QC) nowadays have to meet release criteria (Decristoforo et al. 2017; Elsinga et al. 2010) like a high radiochemical yield (RCY) and radiochemical purity (RCP). RCY is defined as: The ratio of the activity of a specified radionuclide of a specified element after its radiochemical separation or labeling and its activity originally present in the substance undergoing the radiochemical separation or labeling. RCP is defined as the ratio of the activity of a radionuclide in a stated chemical species in a material over the total activity of all species containing that radionuclide in this material (Coenen et al. 2018). RCP measurements are based on specific detection techniques with liquid chromatography (LC). For multicenter trial, validation, standardization and coordination of the release criteria is required, especially because of the use of a variety of (U)HPLC systems. Therefore, in this manuscript we would like to indicate systemic variations in detection, data interpretation and processing in more detail. Some practical aspects are included for specific radiopharmaceuticals; like, limit of detection, threshold and interpretation of impurities. For each subject we explain the needs and the difficulties separately. This overview gives practical examples and should lead to better understanding and is a recommendation for a proper comparison of RCP between different institutes.

### In-line detection of activity/measurement of activity in combination with liquid chromatography

Many data output in radiopharmacy is commonly based on detection of radioactivity, which is usually measured with an in-line detector in liquid chromatography. In-line detection is described in general monographs in the Ph. Eur. 2.2.66 (European Pharmacopeia 9.0 2017a). Briefly, a sample containing a radiopharmaceutical is diluted (if necessary) and injected onto a chromatography system with specified volume, conditions and column as written in accordant standard operating procedure (SOP). Important QC parameters for in-line detection on LC are limit of detection (LOD), limit of quantification (LOQ) and detector linearity (Guide for the elaboration of monographs on radiopharmaceutical preparations 2018). Numerous more parameters influence the activity quantification such as the detector’s crystal dimensions, inner diameter and length of tubing (flow cell) used, position of tubing with respect to crystal (in front of or through crystal) and distance of tubing with respect to crystal. Counting efficiency can be increased by coiling of tubing, however this will reduce peak separation. Overall there will be a balance between sensitivity, separation and resolution (European Pharmacopeia 9.0 2017a).

RCP measurements of radiopharmaceuticals are based on radioactivity detection by detectors and adhering equipment and software, quite often of different brands. Specific detectors such as GABI, MIRA (Raytest), osprey (Canberra), Bpad (Brightspect) and Flow-RAM (LABLOGIC) are used. Moreover, detection is performed by specific scintillator crystals i.e. CeBr, NaI and BGO (Bi_4_Ge_3_O_12_).

Important parameters which can influence radioactivity detection for the in-line measurement and thus have to be validated and standardized are count rates and efficiency, yield-differences for specific energies (keV), response time electronics, hardware/software sampling rates, intrinsic noise, distance between radiodetector and sample, flow cell length, internal diameter and volume. More details and specific influence on different parameters are described by de Zanger et al. (Manuscript in preparation).

### Settings for validation and optimal measuring of RCP

Radiochemical yield is the most important, and the easiest to measure, validate and standardize. In development state of radiopharmaceuticals, RCP is as important but much more difficult to validate and standardize. Determination of the RCP is a quantitative determination of individual impurities. Important parameters here are threshold settings and conditions for integration of peak areas (processing method).To process such radiochromatogram profiles the disregard limit is dependent on the method and related to the LOD and LOQ which will be more explained later. Identification of the different radiopharmaceuticals and related impurities on radiochromatogram profiles is based on retention times of the peak. Peaks areas are used to quantify impurities and are obtained by direct integration of the detector signal using commercially available software.

RCP is well defined in general Ph. Eur. ((chapter 5, Radiopharmaceutical preparations)) (European Pharmacopeia 9.0 2017a; European Pharmacopeia 9.0 2017b) and is as mentioned before, one of the release criteria of radiopharmaceuticals. Therefore it is important to have a validated separation method, which has optimal separation between different (radio)chemical forms (radioactive impurities) other than the original intact labeled radiopharmaceutical.

Important parameters which also have to be considered are accuracy, precision (repeatability and intermediate precision), specificity, linearity and range. These items are well defined in “Guide for the elaboration of monographs on radiopharmaceutical preparation” (chapter 2, EDQM 2018) (Guide for the elaboration of monographs on radiopharmaceutical preparations 2018).

To ensure accuracy in determination of RCP, sample could be spiked by i.e. adding known amounts of impurities to a sample.

Radiochemical impurities may originate from:

- Radionuclide impurities;
- Radiolabeling procedure;
- Incomplete preparative separation;
- Chemical changes of the intended molecule during storage

Individual monographs often include limits of relevant potential radiochemical impurities, including isomers. For multicenter clinical trials, before a specific radiopharmaceutical can be applied clinically, methods of radiolabeling and concordant QC to be performed has to be validated in a radiopharmacy. Also reproducibility which expresses the precision between laboratories are of importance specifically for collaborative studies. General methodology of validation is described in ICH Q2 (R1).

### Smoothing of HPLC data

Despite all difference in hardware there are also multiple options for smoothing the data. This could be done either by changing the sampling frequency (Hz) or by using a specific smoothing algorithm afterwards. (i.e. mean or Savitzky-Golay). Savitzky and Golay (1964) is a type of smoothing which can be applied in a set of digital data points. Smoothing can be applied to increase signal-to-noise ratio without distorting the signal. Data points will be fit with a low degree polynomial by the method of linear least squares. The mean smoothing is a more basic type of smoothing and is simply based on the mean of the points taking into account. Here we made and comparison of these specific algorithms to show influences in RCP outcomes (see Figs. 3, 4, 5).

### Interpretation of chromatograms

#### Background correction and threshold

**Fig. 1:**
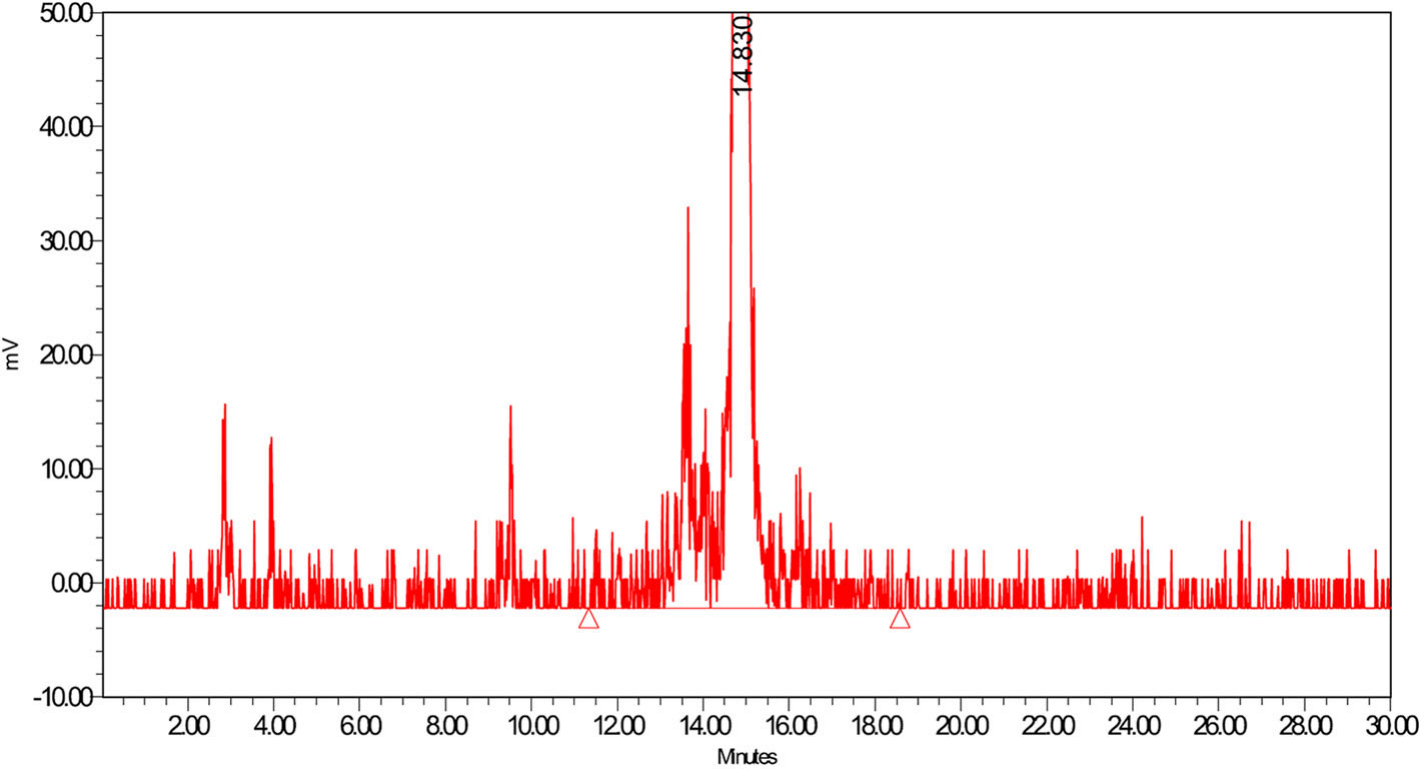
Chromatogram example: factors which are very important to be taken into account are i.e. half-life, background correction

We also investigated the influence of setting a threshold. Threshold specifies the liftoff and touchdown value (minimum rate of change of the detector signal) for peak detection (Fig. 1).

#### Limit of detection (LOD) and limit of quantification (LOQ)

As defined in Ph. Eur. 2.2.66. (European Pharmacopeia 9.0 2017a): LOD is an estimation of the background signal and its standard deviation, The LOD is usual considered to be background plus 3 times the standard deviation of the background signal. This also counts for LOQ, this value is used particularly for the determination of impurities. Practically the LOQ is usually considered to be background signal plus 10 times the standard deviation of the background signal.

## Materials and methods

### Radiolabeling

Radiolabeling for three radiopharmaceuticals and QC were performed according accordant SOP, briefly:

- DOTA-Bombesin containing kit (25 μg) was connected to a 1110 MBq ^68^Ga generator (ITG, Germany) and 5 mL of 0.05 M HCl was eluted into the vial and placed in a dry bath for 20 min at 85 °C. After labeling vial was taken out and cooled down to room temperature (RT) for 10 min. For HPLC QC, aliquot was taken by 1 mL syringe and added to a 100 μL DPTA solution.
- DOTA-CCK containing kit (50 μg) was defrosted and [^111^In]InCl_3_ in 0.1 M HCl, (Mallinckrodt Medical, The Netherlands) was added. ~ 240 MBq (< 0.5 mL) was placed in a dry bath for 20 min at 90 °C. After labeling vial was taken out and cooled down to RT for 10 min. For HPLC QC aliquot was taken by 1 mL syringe and added to a 100 μL DPTA solution.
- 100 μg DOTA-PSMA was dissolved in 0.5 mL containing 4 mg gentisic acid, subsequently 6 mL of 0.04 M acetic acid was used to dissolve 13 mg of sodium ascorbate and 31 mg of sodium acetate-tri hydrate. Two solutions were combined to have a total volume of 6.5 mL. To this solution [^177^Lu]LuCl_3_ (80 GBq/mL, AAA/IDB Holland, the Netherlands) was added (4 GBq ~ 50 μL). Labeling was then initiated by heating the vial at 80 °C for 20 min. Labeling was stopped by cooling down the reaction mixture to RT. After labeling, solution containing sodium chloride, DTPA and ethanol was added to the reaction vial (11 mL). This solution was created by adding 1.2 mL of ethanol (≥99%) to the sodium chloride/DTPA solution (10 mL) delivered with the hardware and reagents kit (Scintomics, Germany). For HPLC QC aliquot was taken by 1 mL syringe.

The RCP of [^68^Ga]Ga-Bombesin, [^111^In]In-CCK and [^177^Lu]Lu-PSMA were measured by HPLC (see Table 4). Gradients were used as described in Tables 1, 2 and 3 using a flow of 1 mL/min.

**Table 1:** Gradient HPLC profile used for [^68^Ga]Ga-Bombesin (Aeris PEPTIDE XB-C18, 3.6 μm 150 × 4.6 mm column) Phenomenex

Time (min)	0.1% formic acid (%)	ACN (%)
0–2	85	15
2–9	60	40
9–11	60	40
11–11.5	0	100
11.5–13	0	100
13–13.1	85	15
13.1–15.5	85	15

**Table 2:** Isocratic HPLC profile used for [^111^In]In-CCK (Kinetex C18, 5 μm 150 × 4.6 mm) Phenomenex, Column oven 40 °C

Time (min)	0.1% TFA (%)	ACN (%)
0–20	75	25

**Table 3:** Gradient HPLC profile used for [^177^Lu]Lu-PSMA (Symmetry C18, 5 μm 150 × 4.6 mm) Waters

Time (min)	0.1% TFA (%)	Methanol (%)
0–20	85	15
20–25	0	100
25.00–25.01	85	15
25.01–30	85	15

### Background correction, LOD, LOQ and threshold

**Fig. 2:**
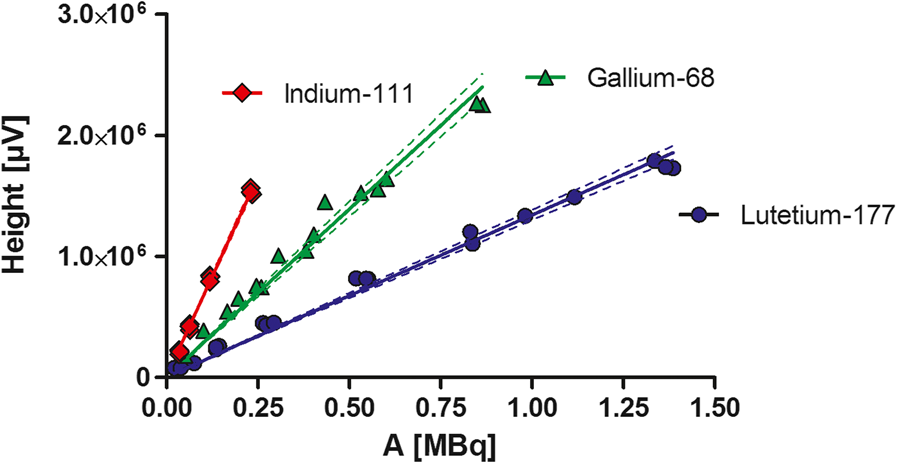
Linearity for Indium-111, Gallium-68 and Lutetium-177 radionuclides determined on used HPLC-radio detector. Pearson R^2^ for Indium-111: 0.996, Gallium-68: 0.972, and Lutetium-177: 0.9885 respectively

Background activity measurements were performed with the shielded in-line detection system by performing a blanc injection (30 min run) during this run the background signal was recorded. This data resulted in a mean background value. Background value for each radionuclide was measured individually. Energy windows were set on their specific energy for used nuclides. LOD and LOQ of Indium-111, Gallium-68 and Lutetium-177 measured by HPLC-radiodetector (see Table 4, Institute 1) were determined by injecting a known amount of [^111^In]In, [^68^Ga]Ga or [^177^Lu]Lu-labeled DOTA-peptide. Correlation of activity (MBq) vs peak height (μV) was obtained by plotting the data into a graph and a linear regression line was drawn through the point of the values obtained by injection of the different activities (Fig. 2). Based on obtained background values, a threshold of 400 μV was introduced to discriminate between a peak and background signal. As described earlier, here we stated that background signal plus 3 times standard deviation is LOD and 10 times for LOQ.

**Table 4:** HPLC-setting for measurements of three different institutes

	Institute 1	Institute 2^a^	Institute 3^b^
HPLC system	Alliance 2695	Thermo fisher ultimate 3000	HPLC dionex ultimate 3000
Radiodetector setup	NaI(Tl) with Osprey-DTB	Raytest GABI	Raytest GABI
Flowcell [μL]	1.3	5	5
Response time/sampling rate (point/Sec)	1	unknown	5
Crystal size [inch]	1	2	2
Crystal material	NaI	NaI	NaI
Software	Empower 3	Chromeleon	Chromeleon 7.1
Response time electronics [s]	~ 0.5	3	7
Type of smoothing	none	unknown	unknown

^a^ and ^b^ see legend Fig. 4

### Smoothing algorithms and RCP outcome

Here we made an overview of two different type of smoothing applied to three different radiopharmaceuticals: [^68^Ga]Ga-Bombesin, [^111^In]In-CCK and a [^177^Lu]Lu-PSMA analog. The two applied smoothing types were (Savitzky and Golay 1964) and mean.

**Fig. 3:**
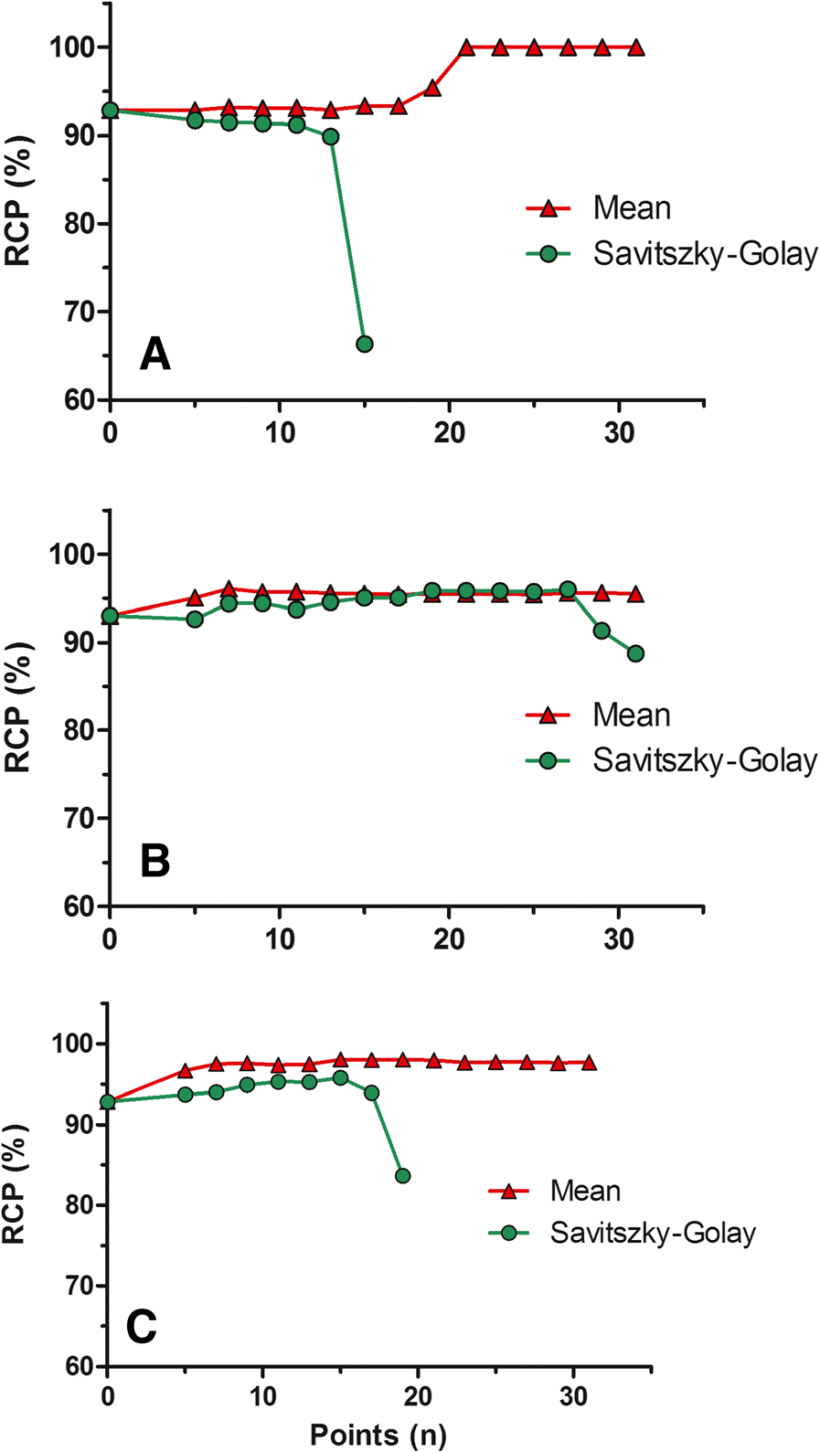
**a**-**c** Overview of 3 radiopharmaceuticals (**a**: [^68^Ga]Ga-Bombesin, **b**: [^111^In]In-CCK and **c**: [^177^Lu]Lu-PSMA) analyzed with 2 different types of smoothing. Peak width was kept constant. Further analyzing conditions are as stated in legend Fig. 5, no threshold was used for analyzing as shown analyzing method E/G

Amount of data points within the smoothing interval were manually defined within the software, over a range of 5–31 data points (Empower3, Waters, Etten-Leur, Netherlands). The radioactive signal sampling rate (using Satin-1 and 2) is 1–2 points per second. Influence of the amount of data points on the obtained RCP was investigated and plotted (Fig. 3).

**Fig. 4:**
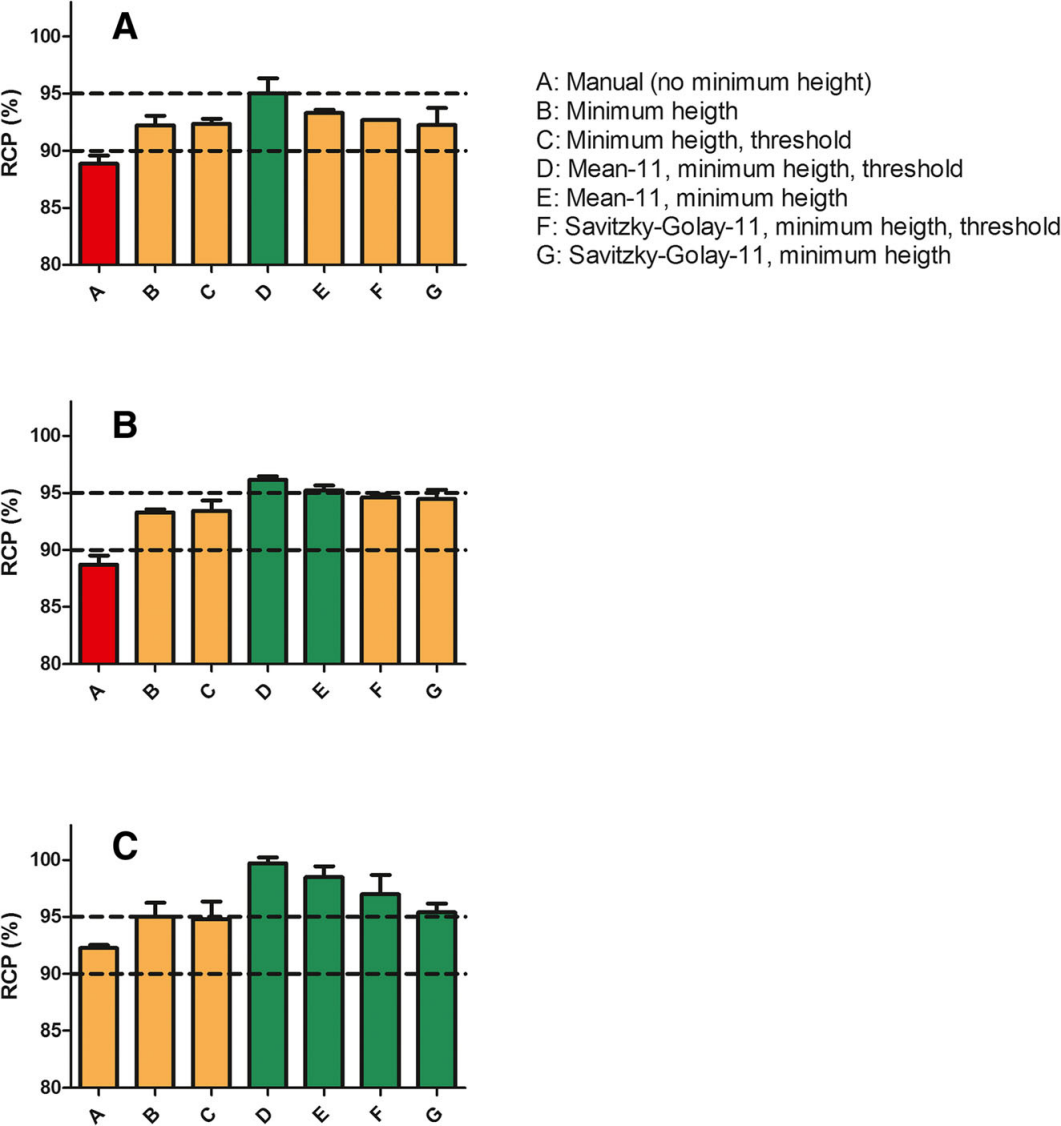
**a**-**c** Overview of 3 radiopharmaceuticals (**a**: [^68^Ga]Ga-Bombesin, **b**: [^111^In]In-CCK and **c**: [^177^Lu]Lu-PSMA) analyzed on 7 different ways. Dotted lines express 90% and 95% RCP limits. Red bars represent values below release criteria, orange bars values between 90 and 95%, green bars above 95%. Minimum peak height was dependent on nuclide (A: 7500 μV, B: 14000 μV, C: 8000 μV). Threshold was at 400 μV, except for condition A. Peak width was kept constant. For all smoothing conditions 11 points were taken into account, this was based on result shown in Fig. 2. RCP was also obtained at other institutes using same kit batches and SOP resulted in a RCP of 96.7%* ([^68^Ga]Ga-Bombesin) and 95.7% and 94.4%** ([^111^In]In-CCK) (for parameters see Table 4)

Obtained RCP results were compared to results from other institutes. In Table 4 an overview is given on the used equipment in each center, with the most important parameters. Results obtained by the three different HPLC systems were compared (legend, Fig. 4). Radiopharmaceuticals were made by using the same labeling SOP.

## Results

### Background correction, LOD and LOQ

A significant difference in background was measured between used radionuclides (Table 5). Based on the linear equation of lines (Fig. 2), LOD and LOQ were measured and calculated peak heights were transferred into activity units (kBq) (Table 5).

**Table 5:** Overview of LOD and LOQ for three used radionuclides

Nuclide	Background (kBq)	LOD (kBq)	LOQ (kBq)
Gallium-68	1.0 ± 0.1	1.3	1.9
Indium-111	0.7 ± 0.1	1.0	1.8
Lutetium-177	3.7 ± 0.3	4.6	6.6

### Smoothing algorithms and RCP outcome

**Fig. 5:**
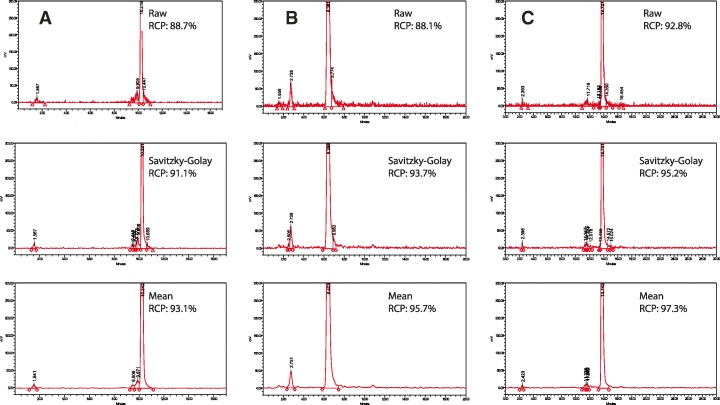
**a** Bombesin-analog labeled with Gallium-68. Chromatogram was analyzed using different type op smoothing. Also manual integration was applied. Difference in RCP is ~ 4.4%. Smoothing algorithm was applied using 11 data points. Minimum peak height was set on 3 times background. RCP from chromatograms were taken into account in Fig. 5a. (A: manual (raw), E: mean, G: Savitszky-Golay. **b** CCK-analog labeled with Indium-111. Chromatogram was analyzed using different type of smoothing. Also manual integration was applied. Difference in RCP is ~ 7.6%. Smoothing algorithm was applied using 11 data points. Minimum peak height was set on 3 times background. RCP from chromatograms were taken into account in Fig. 5b. (A: manual (raw), E: mean, G: Savitszky-Golay. **c** PSMA-analog labeled with Lutetium-177. Chromatogram was analyzed using different type op smoothing. Also manual integration was applied. Difference in RCP is ~ 4.5%. Smoothing algorithm was applied using 11 data points. Minimum peak height was set on 3 times background. RCP from chromatograms were taken into account in Fig. 5c. (A: manual (raw), E: mean, G: Savitszky- Golay

A comparison of 3 radiopharmaceuticals, 3 different peptides labeled with 3 different radionuclides was made. Outcomes of all used integration parameters are summarized in Fig. 3a-c. Visual samples are given in Fig. 5a-c. As shown in Fig. 3a-c influence on RCP is depended on amount of data point and as well on type of smoothing used. For Savitszky-Golay, < 12 points dramatically influence RCP outcome. Additionally influence of smoothing on RCP is also dependent on radiopharmaceutical, this is highly likely dependent on peak shape (Fig. 5a-c).

## Discussion

### In-line detection of activity/measurement of activity in combination with liquid chromatography

In the introduction we gave some examples of equipment used for in-line detection of activity. Many of those detectors and scintillation cristals are in use for specific reasons like high count-rate efficiencies for specific type radionuclide (γ,β,α) or because of detecting specific energies (high vs low energies). Data outcome of different combinations of detectors are often compared to each other without well-defined Quality Assurance / Quality Control (QA/QC) procedures and comparison of these detector systems. Therefore intermediate precision and reproducibility measurements are highly recommended (Guide for the elaboration of monographs on radiopharmaceutical preparations 2018). Overall, when validation is performed in the correct way these phenomena should not occur.

### Settings for validation and optimal measuring of RCP

Determination of the RCP is a quantitative determination of individual impurities. Ph. Eur. (chapter 5, Radiopharmaceutical preparations) also states that: “In principle, any method of analytical separation may be used in the determination of RCP” (European Pharmacopeia 9.0 2017b). Moreover, RCP is an arbitrary unit that is defined as the amount of radioactivity measured in the main peak (radiopharmaceutical) vs the total amount of radioactivity injected/measured. Thus, the RCP depends on the ability to measure radioactivity and could be influence by all factors described earlier. Assuming that all factors for proper activity detection are well defined, there are multiple other factors like injection volume, injection matrix, eluent, gradient, flow, temperature etc. which influencing a LC system and therefore the RCP value. Al those factor should be check, validated and standardized and defined in accordant SOP. More effort should be taken to validate and standardize methods between institutes. Therefore we should use the so called intermediate precision which expresses variation within laboratories: different days, analysts equipment and more (Guide for the elaboration of monographs on radiopharmaceutical preparations 2018)*.* For accuracy check between pharmacies, it is common to send around specific standards, so called “ring trial”. These outcomes are gathered and comparison is made. For radiopharmacy there are some difficulties like short half-lifes and radiolysis after labeling. In our arguable opinion, till nowadays there are no proper alternatives. Nevertheless, for comparison of equipment for in-line radioactive detection between pharmacies this procedure could be useful. Sending around a “hot liquid” is desirable. This is a ready to use radiopharmaceutical which can be distributed within a certain amount of time, especially developed for i.e. clinical trials.

### Smoothing of HPLC data

Smoothing algorithms are widely used to process data outcome. In our opinion, drawback of these smoothing’s is that it can influence background signal and suppress high frequent noise. This makes it more difficult to discriminate a peak from the actual background signal. A signal-to-noise ratio between 3:1 and 2:1 is generally accepted (Guide for the elaboration of monographs on radiopharmaceutical preparations 2018). As shown with our data (Fig. 3), the amount of data points taken into account is crucial, the more points taken the more suppression of the noise and therefore influence on HPLC spectra will increase. For comparison this need to be well defined.

### Background correction and threshold

Despite HPLC-detectors are well shielded, during analyses of a specific radiotracer, there will always be additional background, this is energy dependent (KeV) thus also nuclide specific. Therefore we strongly recommend comparison of i.e. HPLC equipment and output even when the same brands are used. A practical example by standardize the shielding and thickness, and geometry of activity measurements.

### Interpretation of impurities

According to the Ph. Eur 2.2.46., when a specific impurity contain < 1% of total peak area on the chromatogram, no qualification is needed (European Pharmacopoeia 9.0 2017). Unfortunately, some difficulties arise when using in-line radioactive measurements, for instance interpretation of peak tailing (European Pharmacopoeia 9.0 2017; Zhang et al. 2014). Additionally, radio-peptides will be randomly damaged by radicals. These radicals are mainly formed by the radiolysis of H_2_O (de Blois et al. 2013; Garrison 1987; Jay-Gerin and Ferradini 2000; Jonah 1995; Slegers and Tilquin 2005; Swiatla-Wojcik and Buxton 2005). Chromatograms of radiolysed radiolabeled products show up as smears because of this. For releasing a product, all signal above minimum peak height, including the hole smear, should be taken into account for determination of final RCP.

### Multicenter standardization

As stated in Ph. Eur. individual monographs include limits of relevant potential radiochemical impurities, including isomers. Because many radiopharmaceuticals are not registered, individual monograph of those simply do not exist. In these occasions general monograph have to be applied. Therefore, harmonization and comparison of equipment before starting an multicenter trial becomes even more important with special focus on the comparison of HPLC equipment and i.e. the applied SOP’s but also HPLC-chromatogram processing methods.

Additionally there are two ways of processing data, using the traditional integration or the Apex Track. Our results are based on the traditional integration, to our knowledge for radiopharmaceuticals no comparison is performed between both integrations methods neither specified in any SOP.

#### Overall recommendations on measurement of RCP for radiopharmaceuticals

As mentioned earlier for accuracy a ring trial is an applied method for comparison of specific measurements between pharmacies. This would be also desirable with radiopharmaceuticals before start of a multicenter trial and obtained values should be compared. In this case the chemical properties of the pharmaceutical are not of importance, only the outcome of measurement of the activity. A difficulty will arise when radiopharmaceuticals with a short lived isotope are used. In this case another isotope with a longer half-life would be more appropriate, then all settings and setups can be compared in advance and the range of outcome can be discussed.

Nowadays vendors have a preference for a so called “hot liquid” (Chakraborty et al. 2018; de Blois et al. 2014). For these specific radiopharmaceutical this has the advantage that many discussions and comparison can be avoided. For standardization, validation and comparison of equipment and procedures, distribution of a hot liquid with longer half-life’s will be a good option, specifically for centers involved in multicenter trials. From this data output, RCP values can be obtained and can be used for comparison.

## Conclusion

Many parameters can influence final output of RCP. Measurement of RCP is easily influenced even when many precautions are applied. To be able to compare RCP between institutes, comparison of (U)HPLC-equipment is mandatory. For this the Guide for the elaboration of monographs on radiopharmaceutical preparations is very useful (Guide for the elaboration of monographs on radiopharmaceutical preparations 2018). For multicenter trials, we recommend to compare locally determined RCP under validated and standardized conditions of in-line activity detection between institutes for each radiopharmaceutical. Based on repeatability and intermediate precision for multicenter trials, a locally determined range of RCP as release criteria is preferred and recommended.

## Abbreviations

(U)HPLC: Ultra high performance chromatography
EDQM: European Directorate for Quality of Medicine
LC: Liquid chromatography
LOD: Limit of detection
LOQ: Limit of quantification
PSMA: Prostate specific membrane antigen
QA: Quality assurance
QC: Quality control
RCP: Radiochemical purity
RCY: Radiochemical yield
RT: Room temperature
SOP: Standard operating procedure
